# Genome-Wide Identification and Characterization of the Key Genes for Salicylic Acid Biosynthesis in Four Cotton Species

**DOI:** 10.3390/ijms27093936

**Published:** 2026-04-28

**Authors:** Jiaqi Lin, Xin Zhou, Shandang Shi, Xin Li, Manhong Wang, Fei Wang, Liping Zhu, Hongbin Li

**Affiliations:** 1 Key Laboratory of Oasis Town and Mountain-Basin System Ecology of Bingtuan, Key Laboratory of Xinjiang Phytomedicine Resource and Utilization of Ministry of Education, College of Life Sciences, Shihezi University, Shihezi 832000, China; linjiaqi7606@126.com (J.L.); shi_shandang@shzu.edu.cn (S.S.); lihb@shzu.edu.cn (H.L.); 2 College of Life Sciences, Shaanxi Normal University, Xi’an 710119, China; zhouxin8852@163.com; 3 National Key Laboratory of Cotton Biological Breeding and Comprehensive Utilization, Henan University, Kaifeng 475004, China; Aa1329552106@163.com; 4 Department of Poultry Science, Mississippi State University, Starkville, MS 39762, USA; mw2911@msstate.edu

**Keywords:** cotton, salicylic acid synthase gene family, fiber development

## Abstract

Cotton, as a globally significant economic crop, is intricately regulated in its growth and development by the key genes for SA (Salicylic acid) biosynthesis. In the present study, a systematic analysis of genes related to SA biosynthesis was conducted across four cotton species, leading to the identification of 70 genes. Specifically, the tetraploid species *Gossypium hirsutum* and *G. barbadense* were found to harbor 22 and 23 genes, respectively, representing a substantial expansion compared to the 12 and 13 genes identified in the diploid progenitors *G. arboreum* and *G. raimondii*. Comprehensive characterization of chromosomal localization, phylogeny, domain architecture, and promoter *cis*-elements revealed a uniform distribution of key genes involved in SA biosynthesis across A/D sub-genomes of tetraploids with extensive interspecific collinearity; whole-genome and segmental duplication act as the dominant drivers for the expansion of this gene family, while partial gene loss following polyploidization results in non-doubled gene copy numbers in tetraploids relative to diploids, which reflects the evolutionary selection for genomic dosage balance. The key genes for SA biosynthesis demonstrate a high degree of conservation in protein sequences, protein structures, and conserved motifs, which constitute the structural basis for the stable maintenance of their core functions in the SA biosynthesis pathway during plant evolution. This is closely related to their core function in the salicylic acid (SA) synthesis pathway and serves as the structural basis for the stable maintenance of gene functions during evolution. Analysis of *cis*-elements revealed that the expression of key genes involved in SA biosynthesis is governed by a complex interplay of phytohormones, stress signals, and transcription factors. Yeast one-hybrid (Y1H) assays confirmed the interaction between the *GhPAL* and *GhICS* gene and predicted candidate transcription factors, specifically the binding of GhWRKY21 to *GhICS2-1* promoter and GhMYB12 to *GhPAL1-2* promoter, thus elucidating their stage-specific regulatory mechanisms in cotton fiber development and reflecting their evolution. This study provides a fundamental basis for investigating the role of the SA signaling pathway in cotton development and offers support for cotton molecular breeding.

## 1. Introduction

Cotton (*Gossypium* spp.) is an important economic crop and the most widely planted natural fiber source worldwide [1]. From a whole-genome perspective, cotton is important because its unique genome structure offers an ideal model for studying polyploid evolution, phenotypic diversity, and adaptive improvement [2]. Cotton has diploid and tetraploid forms, and the tetraploid form was formed about 1.5 million years ago [2,3]. It was formed through hybridization and genome doubling of diploid ancestral species that carry the A genome and D genome [4,5]. Currently, tetraploid upland cotton is the main cultivated cotton species [6]. In recent years, advances in high-throughput sequencing technologies have allowed researchers to obtain reference genomes for several diploid ancestral cotton species and tetraploid cultivated species [7]. These genomes provide useful references for understanding the molecular basis of cotton’s phenotypic diversity, finding beneficial alleles, and guiding molecular design breeding.

Cotton is the most important natural fiber crop in the world, and its economic value mainly comes from mature cotton fibers. These fibers are formed through the differentiation of seed epidermal cells, and this process is also an ideal model for studying plant cell elongation, cell wall synthesis, and polar growth [8,9]. Cotton fiber development is a complex and precise process that goes through four continuous and partially overlapping stages: fiber initiation, fiber elongation, secondary wall thickening, and mature boll opening [10]. During cotton fiber development, fine transcriptional regulatory switches occur between stages [11,12]. These switches directly affect key traits like fiber number, length, strength, and fineness, which determine cotton yield and quality. These switches are also core targets for improving fiber traits in cotton molecular breeding.

Salicylic acid (SA), an important phytohormone, exerts a broad-ranging influence on the regulation of plant growth, development, and responses to environmental stresses [13]. Through the inter-conversion of these different forms, SA participates in the regulation of multiple physiological processes related to growth, maturation, and senescence, thus establishing an integrative connection between developmental programs and stress-responsive signaling networks [14]. Within the plant kingdom, SA biosynthesis occurs predominantly through two well-established routes: the isochorismate synthase (ICS) pathway and the phenylalanine ammonia–lyase (PAL) pathway [15,16]. ICS enzymes catalyze the transformation of chorismate to isochorismate within plastids, which represents a crucial regulatory step in SA production; isochorismate is then transported to the cytosol by EDS5 and further modified by PBS3 to form an unstable intermediate that can be converted into SA either spontaneously or via EPS1 [17,18]. Beyond the ICS pathway, a conserved three-enzyme module (BEBT-BBH-BSE) mediates PAL-dependent SA biosynthesis by converting benzoyl-CoA to SA via benzyl benzoate and benzyl salicylate rather than direct hydroxylation of benzoic acid, and this system is evolutionarily conserved in most crops, including cotton and rice [19,20]. SA signaling activates PR gene expression via the NPR1-TGA activator complex, while NPR3/NPR4 act as SA receptors that form repressive complexes with TGA factors to suppress defense genes under low SA levels [21,22,23].

Salicylic acid (SA) is a classic defense hormone, and recent studies show that it also has an important regulatory function in cotton fiber development. The cotton metabolic regulatory network (CMRN), built by integrating metabolome and transcriptome data, reveals key genes in multiple metabolic pathways during fiber development and provides a systematic framework for studying the roles of SA and related hormones in fiber development [24]. Multiple stages of fiber development involve a complex and highly coordinated hormone regulatory network, in which plant hormones play a core regulatory role [25,26,27]. Notably, the regulatory role of SA in cotton fiber development is closely linked to environmental stress. When cotton plants experience biotic or abiotic stress, SA levels change significantly. By activating the expression of downstream defense-related genes, SA helps balance normal fiber development with enhanced stress resistance. At the same time, this process can indirectly affect fiber yield and quality [28,29].

In the present study, we took a genome-wide approach to identify and analyze the key SA biosynthesis genes across four cotton species—the tetraploids *G. hirsutum* and *G. barbadense*, and the diploids *G. arboreum* and *G. raimondii*. Their phylogenetic relationships, chromosomal distribution, gene structure and conserved motifs, *cis*-regulatory elements, collinearity, and expression profiles were systematically examined, providing an integrated evolutionary and functional overview of the *PAL* and *ICS* genes in cotton. Additionally, Y1H (Yeast one-hybrid) analyses were conducted to experimentally evaluate the regulatory characteristics of selected *GhPAL* and *GhICS* genes. Collectively, these findings provide a fundamental framework for comprehending SA biosynthesis in cotton and may promote future research on hormone-mediated regulation of fiber development and stress adaptation.

## 2. Results

### 2.1. Identification of SA Biosynthesis-Related Genes in Cotton

We identified 70 SA biosynthesis-related genes in four cotton species. The distribution of the 70 genes was as follows: 22 in *G. hirsutum* (2 *GhADAS*, 4 *GhASA*, 2 *GhICS*, and 14 *GhPAL*), 23 in *G. barbadense* (2 *GbADAS*, 4 *GbASA*, 4 *GbICS*, and 13 *GbPAL*), 13 in *G. raimondii* (1 *GrADAS*, 2 *GrASA*, 2 *GrICS*, and 8 *GrPAL*), and 12 in *G. arboreum* (1 *GaADAS*, 2 *GaASA*, 2 *GaICS*, and 7 *GaPAL*) (Table S1). These findings suggest that the SA biosynthesis-related genes are well represented in both diploid and tetraploid cotton species. 4-Amino-4-deoxychorismate synthase (ADCS), Acetylsalicylic acid (ASA), and ICS share the same substrate (chorismate). As core members of the chorismate metabolic node, they control carbon flow and affect ICS-mediated SA biosynthesis. ADCS and ASA can compete for substrate and influence the ICS pathway. Thus, all three are considered genes that regulate SA biosynthesis. In the following analysis, we focused mainly on *ICS*, *ASA*, *ADCS*, and *PAL*.

### 2.2. Physico-Biochemical Property Analysis

A systematic analysis was conducted on the physico-chemical properties of proteins encoded by SA biosynthesis-related genes in cotton genomes (Table S2). The lengths of the predicted proteins spanned from 491 amino acids (GbPAL2-3) to 916 amino acids (GaADCS), with an average length of 684.09 amino acids, suggesting a relatively concentrated size distribution among members of the SA biosynthesis-related genes. The predicted molecular weights ranged between 54.36 kDa (GbPAL2-3) and 102.84 kDa (GbADCS-1). The molecular weights of the vast majority of members were concentrated between 60 and 85 kDa. This relatively homogeneous molecular size distribution reflects the structural conservation of key enzymes in the SA synthesis pathway during evolution, providing a molecular structural foundation for stable SA synthesis at different growth stages and ensuring normal hormone balance maintenance in cotton. The theoretical isoelectric points (pI) fluctuated from 5.49 (GbICS2-3) to 9.09 (GbPAL2-3), with an average pI value of 6.34, implying that the majority of the proteins are acidic, while only a small number of members are alkaline. These proteins exhibit overall weak acidity, which facilitates their solubility in the chloroplast matrix and cytoplasmic environments of cotton leaves, maintains catalytic activity, and ensures accurate subcellular localization. The presence of a few alkaline members may indicate functional differentiation to meet specific demands under particular tissues or stress conditions. Protein stability analysis showed that the instability index (II) of these proteins ranged from 27.53 (GaPAL1-2) to 56.89 (GhICS1-3). Of these, 43 proteins (61.4%) were stable (II ≤ 40) and 27 (38.6%) were unstable (II > 40), with the stable ones being responsible for basal SA synthesis under normal growth conditions and thus ensuring the hormonal balance essential for cotton growth and development, while the unstable counterparts can be rapidly synthesized and activated in response to adverse stress, only to be promptly degraded after fulfilling their biological functions so as to avoid cotton growth inhibition caused by excessive SA accumulation. The aliphatic index (AI) of the proteins ranged from 81.26 KDa (GbICS2-1) to 103.74 KDa (GhPAL2-3), with an average of 92.58 KDa, suggesting good thermal stability potential, enabling them to maintain catalytic activity under non-biological stresses such as high temperatures, which aligns with the temperature fluctuation environment encountered during cotton field growth. The grand average of hydropathicity (GRAVY) values were all negative (–0.382 to –0.095), with an average of –0.217, indicating that all proteins are hydrophilic, guaranteeing efficient dissolution, diffusion, and catalytic function at the synthesis site. Subcellular localization prediction showed that 62 proteins were localized in chloroplasts, 5 in cytoplasm, and 3 in peroxisomes. This indicates that the SA biosynthesis pathway in cotton primarily occurs in chloroplasts, while members distributed in other tissues can rapidly respond to intracellular signals, enabling localized SA supplementation. This is similar to other plants, which provides an important molecular basis for cotton to efficiently cope with abiotic stresses and biotic stresses [30,31,32,33].

### 2.3. Chromosomal Location Analysis of SA Biosynthesis-Related Genes in Cotton Species

Chromosome localization showed that in *G. barbadense*, the 23 genes were distributed across 17 chromosomes, with 12 genes located on the A subgenome and 11 genes on the D subgenome (Supplementary Figure S1A). In *G. arboreum*, the 12 genes were mapped to 9 chromosomes, among which chromosomes Chr05 and Chr10 each contained two genes, while the remaining chromosomes harbored one gene each (Supplementary Figure S1B). In *G. hirsutum*, 10 genes were located in the A subgenome, and 3 genes were mapped to unanchored scaffold sequences (scaffold1898, scaffold3390, scaffold4407). Based on sequence homology and collinearity analysis, these scaffold sequences were preliminarily anchored to chromosomes A07, A12, and D10. The remaining 19 genes were distributed on 15 chromosomes, among which 9 genes were located in the D subgenome (Supplementary Figure S1C). In *G. raimondii*, 12 genes were mapped to 9 chromosomes. Among them, chromosomes Chr05, Chr05, Chr10, and Chr11 each contained 2 genes, and the remaining chromosomes each carried 1 gene (Supplementary Figure S1D). The total number of genes in tetraploid cotton varieties was significantly higher than that in diploid cotton varieties, suggesting that gene duplication drove family expansion, while the differences in gene numbers between diploid and tetraploid subgenomes, as well as the deletion of some chromosomal loci, indicate that gene loss events occurred after polyploidization. Therefore, in the four cotton species, the chromosomal distribution patterns indicate that gene deletions and duplications within the key genes in SA biosynthesis occur across cotton species.

### 2.4. Phylogenetic Analysis of SA Biosynthesis-Related Proteins

**Figure 1:**
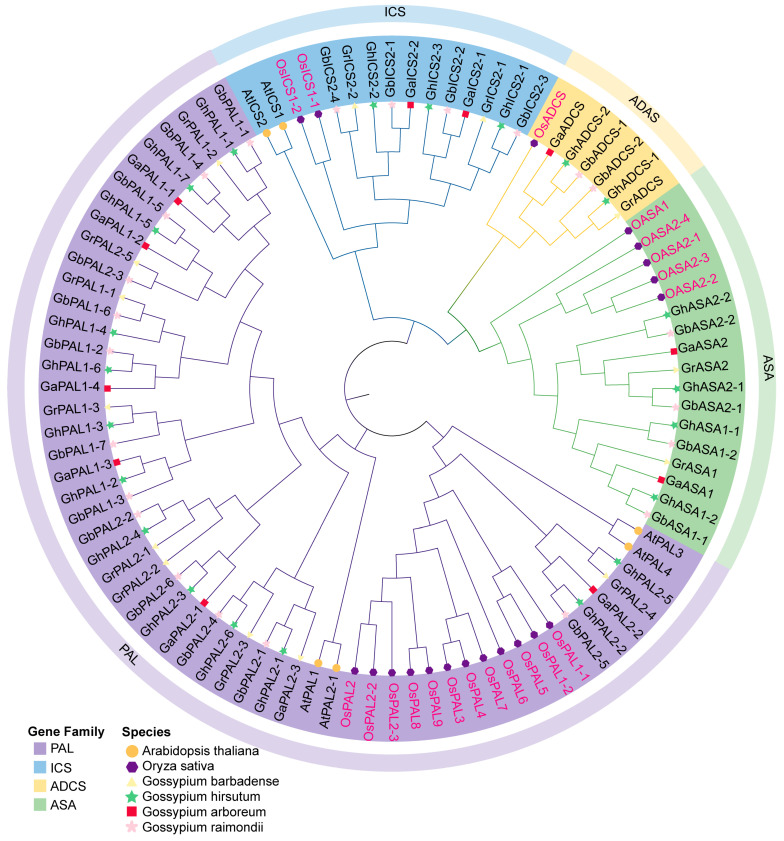
Phylogenetic analysis of SA biosynthesis-related genes. The Isochorismate synthase (ICS), 4-Amino-4-deoxychorismate synthase (ADAS), Acetylsalicylic acid (ASA) and Phenylalanine ammonia-lyase (PAL) protein sequences were obtained from *G. hirsutum* (Gh), *G. arboreum* (Ga), *G. barbadense* (Gb), *G. raimondii* (Gr), *O. sativa* (Os) and *A. thaliana* (At). The phylogenetic tree was generated using MEGA X software (v10.1.8) with the Maximum Likelihood (ML) method, and bootstrap values were based on 1000 replicates. The phylogenetic tree was constructed using the best-fit Jones–Taylor–Thornton (JTT) model with Gamma distribution (+G) and default parameters in MEGA X. Colored outer strips and branches in the circular tree indicate different gene families, and the markers at the end of branches correspond to different species, as detailed in the embedded legend.

A phylogenetic tree was constructed employing the PAL and ICS protein sequences derived from cotton species, rice and *A*. *thaliana* (Figure 1). The protein sequences of 90 SA biosynthesis-related genes from six species were classified into four subfamilies: PAL, ICS, ADCS, and ASA. Among them, the ADCS and ASA subfamilies are absent in *A*. *thaliana*. We observed from the phylogenetic analysis that in the four subfamilies, the ADCS subfamily comprises 7 members, making it the smallest in terms of membership. The ASA subfamily has 17 members, while the ICS subfamily includes 15 members. The PAL subfamily is the largest group, comprising a total of 51 PAL members. Additionally, this study introduces the model crop rice (*Oryza sativa*) for comparative analysis. In rice, 2 *ICS*, 1 *ADCS*, 5 *ASA*, and 12 *PAL* genes were identified. In addition, phylogenetic analysis of SA biosynthesis-related genes showed that cotton and rice genes formed distinct, separate clades, indicating clear evolutionary divergence between dicot and monocot lineages.

### 2.5. Conserved Motifs and Structural Features of PAL and ICS Proteins

**Figure 2:**
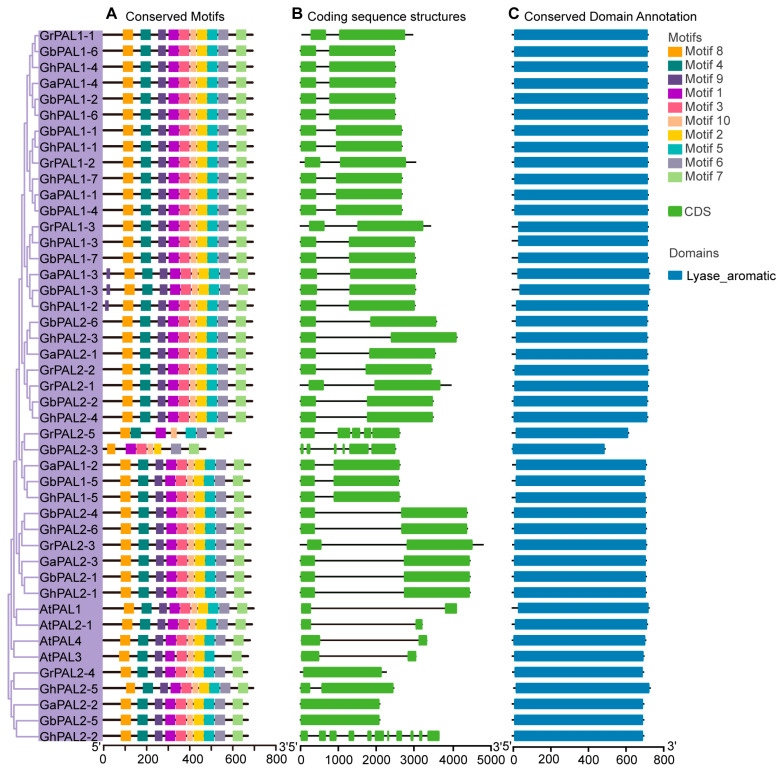
Analysis of conserved motifs, gene structure, and conserved functional domains of PAL from *G. hirsutum*, *G. arboreum*, *G. barbadense*, *G. raimondii* and *A. thaliana*. (**A**) Conserved motif distribution of PAL proteins. (**B**) Coding sequence structures of *PAL* genes. Green boxes indicate coding sequences (CDSs), and black horizontal lines indicate introns. The length of CDSs and introns is proportional to the actual sequence length, with the horizontal axis representing the sequence position in base pairs (bp). (**C**) Conserved functional domain annotation of PAL proteins. Sequence orientation from 5′ to 3′.

Conserved motif and domain analyses indicated that members within each SA subfamily displayed highly analogous motif compositions and domain architectures. The PAL subfamily consistently encompassed the conserved Lyase_aromatic domain, indicating significant functional conservation within this subfamily (Figure 2). This domain was annotated using the Pfam database and NCBI CDD. The Lyase_aromatic domain is a characteristic catalytic domain of PAL proteins and belongs to the aromatic amino acid lyase superfamily. It is noteworthy that GrPAL2-5 and GbPAL2-3 are truncated sequences with incomplete conserved motifs and functional domains.

**Figure 3:**
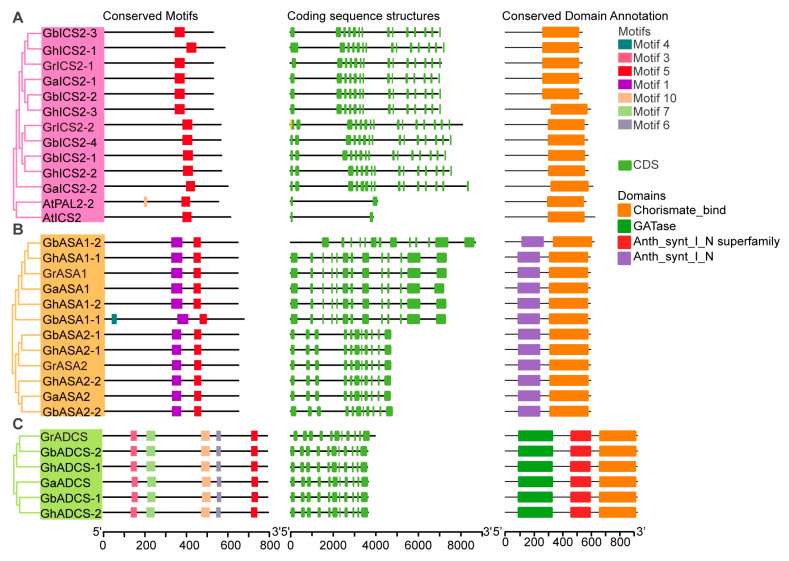
Sequence organization and structural feature analysis of key gene families involved in SA biosynthesis from *G. hirsutum*, *G. arboreum*, *G. barbadense*, *G. raimondii* and *A. thaliana* (At). (**A**) Structural analysis of ICS members. (**B**) Structural analysis of ASA members. (**C**) Structural analysis of ADCS members. From left to right, the three columns represent conserved motif distribution, coding sequence structure, and conserved functional domain annotation. Sequence orientation from 5′ to 3′.

ADCS, ASA, and ICS are core functional members in the SA biosynthesis pathway mediated by the ICS pathway. In ADCS, ASA and ICS proteins, the Chorismate_bind domain was detected, which is indispensable for the binding of chorismate, a precursor in the biosynthesis of SA (Figure 3). However, the Anth_synt_I_N domain was detected in both the ADCS and ASA subfamilies (Figure 3B,C). In addition, the ADAS protein also contains a GATase domain.

However, in the ADCS and ASA subfamilies, conserved motifs and domain architectures were also identified, further supporting the functional conservation of these subfamilies (Figure 3B,C). In ADCS and ASA proteins, the Lyase_I_like and Anth_synt_I_N domains were detected, both of which are critical for enzymatic activity and the regulation of biosynthesis. These results indicate that the SA biosynthetic pathways in these subfamilies are highly conserved with limited divergence, underscoring the essential roles of these proteins in plant metabolic processes.

### 2.6. Cis-Promoter Element Analysis of SA Biosynthesis-Related Genes

**Figure 4:**
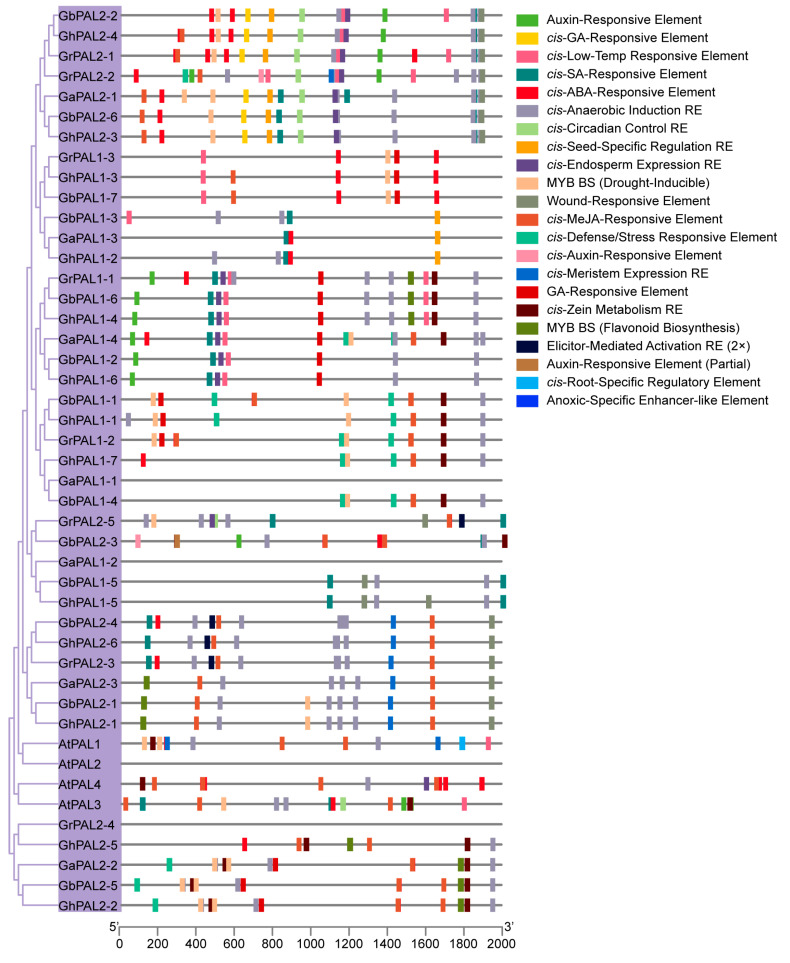
Analysis of *cis*-regulatory elements in SA biosynthesis-related gene promoters: *PAL* promoters in cotton and *Arabidopsis.* The distribution of hormone-responsive, stress-responsive, and tissue-specific *cis*-regulatory elements in the 2000 bp promoter regions upstream of the translation start site (ATG) of *PAL* genes from cotton species, including *G. hirsutum*, *G. barbadense*, *G. arboreum*, *G. raimondii* and A. thaliana, is visualized. Different colored boxes represent distinct types of *cis*-elements, including auxin-, ABA (Abscisic Acid)-, GA (Gibberellin)-, SA-, and MeJA (Methyl Jasmonate)-responsive elements, as well as low-temperature, wound, defense/stress, and tissue-specific regulatory elements. The 5′ and 3′ ends of the promoter sequences are indicated on the *x*-axis.

**Figure 5:**
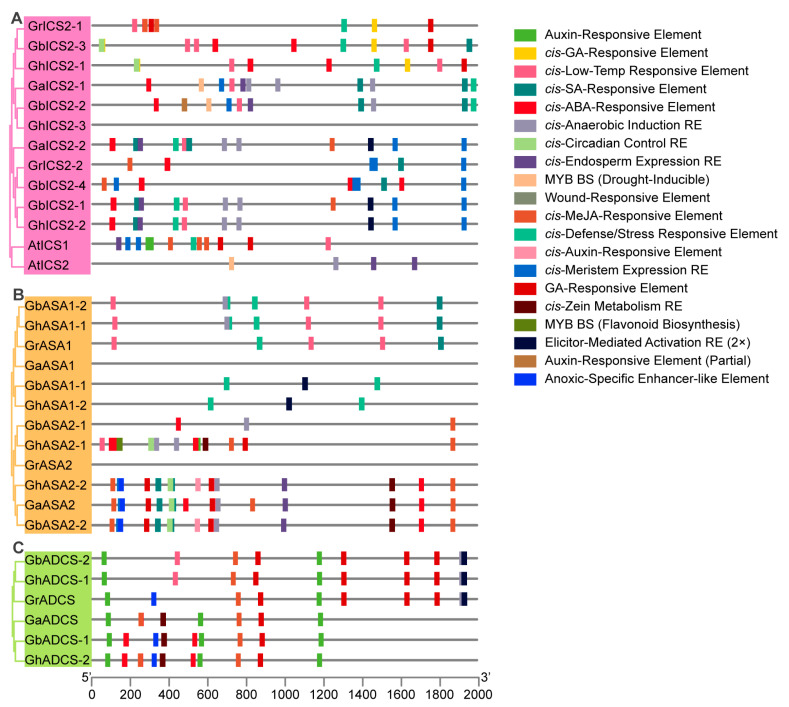
Analysis of *cis*-regulatory elements in the SA biosynthesis-related gene promoters: (**A**) *ICS1/2* promoters in cotton and *Arabidopsis.* (**B**) *ASA1/2* promoters in cotton. (**C**) *ADCS* promoters in cotton. It shows the distribution of hormone and stress-responsive *cis*-elements within the 2000 bp promoter region upstream of the translation initiation site (ATG). Squares of different colors represent different types of *cis*-elements, including auxin, GA, ABA, SA, MeJA, low-temperature, wounding, and defense/stress-responsive elements, as well as tissue-specific and light-responsive elements. The *x*-axis represents the 5′→3′ position of the promoter sequence relative to the translation initiation site.

We observed that the promoters of the genes related to SA biosynthesis were enriched with motifs responsive to phytohormones and abiotic stresses. Such regulatory features play a pivotal role in controlling developmental processes and enhancing stress tolerance. Promoter analysis revealed *cis*-elements in stress and hormone responses: SA, Abscisic Acid (ABA), Methyl Jasmonate (MeJA), auxin, low-temperature, drought, and wound responses within the cotton SA biosynthesis-related gene sequences (Figure 4 and Figure 5).

To be specific we identified *cis*-elements responsive to both SA and ABA within the promoters of the genes related to SA biosynthesis. The presence of these elements points to a function in stress adaptation, particularly against drought and cold stress. We also identified MeJA-responsive motifs within the gene promoters. The presence of these elements points to a role in modulating defense responses, specifically during wounding and pathogen attack. Moreover, the auxin-responsive elements and GA (Gibberellin)-responsive elements detected in these promoters suggest that SA biosynthesis may also be involved in the regulation of growth and developmental processes in cotton, particularly fiber development. The presence of light-responsive and meristem-responsive elements further indicates the possible participation of these genes in the regulation of growth and differentiation under different light conditions and during meristematic activity.

Overall, these results suggest that SA biosynthesis genes in cotton are highly sensitive to a wide spectrum of environmental and hormonal signals, enabling them to coordinate cotton’s growth, development, and stress responses. Therefore, SA biosynthesis-related genes in cotton contain numerous *cis*-elements responsive to stress and hormone signals in their promoter regions, indicating that these genes may be transcriptionally regulated by diverse environmental and hormonal stimuli.

### 2.7. Collinearity Analysis of SA Biosynthesis-Related Genes

Comparative synteny analysis revealed that SA biosynthesis-related genes display extensive collinearity among *G. arboreum*, *G. hirsutum*, *G. barbadense*, and *G. raimondii* (Supplementary Figure S2). A multitude of orthologous gene pairs were detected between *G. arboreum* (A genome) and the At subgenomes of *G. hirsutum* and *G. barbadense*, as well as between *G. raimondii* (D genome) and the Dt subgenomes of these two tetraploid species, confirming the biparental origin of SA biosynthesis-related genes in tetraploid cotton. Moreover, dense and continuous syntenic associations were noted between *G. hirsutum* and *G. barbadense*, implying a high degree of structural conservation of the key genes for SA biosynthesis subsequent to polyploidization. The majority of the collinear gene pairs were related to interchromosomal or intersubgenomic regions, corroborating whole-genome and segmental duplication as the principal factors driving the expansion of the key genes for SA biosynthesis in cotton.

### 2.8. Expression Landscape of GhPAL, GhICS, GhASA and GhADCS During Cotton Fiber Development

**Figure 6:**
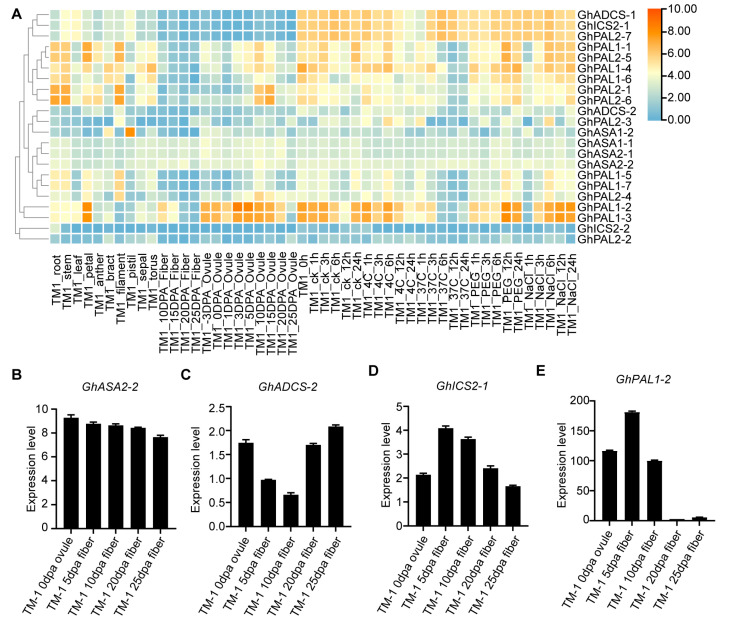
Expression patterns of *GhPAL*, *GhICS*, *GhASA* and *GhADCS* members in various tissues and developing fibers: (**A**) Heatmap showing the expression profiles of selected *GhPAL*, *GhICS*, *GhASA*, and *GhADCS* genes across various cotton tissues (root, stem, leaf, ovule) and fiber developmental stages (5, 10, 15, 20, and 25 dpa). Color scale represents log_2_-transformed relative expression levels (blue = low expression, orange/red = high expression). The genes marked with red emphasis are the key genes of the four types of SA synthetases in *G. hirsutum* that have high expression levels during the fiber development stage. (**B**–**E**) Relative expression of *GhASA2-2*, *GhADCS-2*, *GhICS2-1*, and *GhPAL1-2* in fibers at different developmental stages. Error bars indicate SD (n = 3); dpa, days post-anthesis.

The expression profiles of genes involved in SA biosynthesis in diverse cotton tissues and fibers at different developmental stages are presented (Figure 6). We investigated the expression patterns of genes related to SA biosynthesis in different cotton tissues, during fiber development, and under different stresses (Figure 6A). The results showed that *GhICS2-1* had stable expression across all tissues, stages, and stress treatments, with no clear specificity. *GhICS2-2* showed high expression during fiber and ovule development and responded clearly to cold, heat, salt, and drought. *GhADCS-1* was highly expressed under abiotic stress, while ASA family genes showed relatively stable expression across different tissues, fiber development stages, and stress treatments. *GhPAL1-2* and *GhPAL1-3* were highly expressed in flower organs and during fiber initiation and elongation, and were activated by multiple abiotic stresses. *GhPAL2-5*, *GhPAL2-7*, and other genes showed high expression in roots, stems, and leaves, and during fiber initiation.

Based on the results of heat map analysis, we selected candidate genes with high expression levels during fiber development and further examined the expression patterns of key candidate genes in fibers across different developmental stages (Figure 6B–E). *GhASA2-2*, *GhADCS-2*, *GhICS2-1*, and *GhPAL1-2* displayed distinct expression patterns. *GhASA2-2* and *GhADCS-2* demonstrated relatively stable expression throughout the developmental stages, accompanied by slight increments in fibers at 10 and 20 dpa. In contrast, *GhICS2-1* exhibited a maximal expression level at 10 dpa, whereas *GhPAL1-2* showed its highest expression at the 1 dpa fiber stage.

These findings suggest that the expression of SA biosynthesis genes is differentially regulated across various tissues, and that fibers exhibit dynamic and stage-specific alterations in gene expression during cotton development.

### 2.9. Y1H Assays Identify Transcriptional Regulators of GhICS2-1 and GhPAL1-2

We predicted the upstream transcription factors of *GhICS2-1* and *GhPAL1-2* using the PlantCARE database (https://bioinformatics.psb.ugent.be/webtools/plantcare/html/, accessed on 20 January 2026), and a total of seven potential target genes were screened out. We performed Y1H assays to validate the predictions, testing 2000 bp target promoter fragments in combination with the CDS of candidate genes. We found that GhWRKY21 associates with the *GhICS2-1* promoter, and GhMYB12 was shown to bind the *GhPAL1-2* promoter. After analyzing the cis-elements of *GhICS2-1* and *GhPAL1-2*, we found a WRKY binding site (W-box) on *GhICS2-1* and a MYB binding site on the promoter of *GhPAL1-2* (Supplementary Figure S3). Thus, we propose that the transcription factors directly bind to these sites and interact. We also analyzed the transcriptome data after SA treatment. After SA treatment, *GhICS2-1* expression increased, but GhWRKY21 expression decreased. This suggests that GhWRKY21 may repress *GhICS2-1*. Meanwhile, both *GhPAL1-2* and GhMYB12 expression decreased after SA treatment, suggesting that GhMYB12 may activate *GhPAL1-2* (Supplementary Figure S4).

## 3. Discussion

Salicylic acid (SA) is a plant phytohormone recognized for its involvement in plant defense mechanisms and responses to stress. The core biosynthetic pathways of SA have been well documented in model plants, with *ICS* and *PAL* being key encoding genes, while *ASA* and *ADCS* function as crucial upstream components in the shikimate pathway that supply the common precursor chorismate for SA biosynthesis [34,35]. Previous cotton research has confirmed the important role of SA in regulating growth development and stress resistance [36,37]. This study conducted a systematic genome-wide identification and evolutionary analysis of four key genes (*ICS*, *PAL*, *ASA*, and *ADCS)* involved in SA biosynthesis in four representative cotton species. The research results enrich the understanding of genes related to SA biosynthesis in cotton and make an important contribution to the literature research on this important plant hormone family.

We traced the evolutionary fate of the genes related to SA biosynthesis across species, highlighting the impact of polyploidization. We compared the gene numbers in cotton with those in rice (Figure 2). The numbers of *ICS* and *ADCS* genes in rice are similar to those in diploid cotton. In tetraploid cotton, the numbers of *ASA* and *PAL* genes are higher than in diploid cotton and rice, which is a normal result of polyploidization rather than a gene family expansion [38,39,40]. The cotton genome harbors 70 genes related to SA biosynthesis, a number that far exceeds the gene complements of other species. Taking the PAL as an example, diploid cotton contains 7–8 *PAL* genes, while tetraploid cotton has 13–14 *PAL* genes, demonstrating significant expansion compared to the 4 *PAL* genes in A. thaliana. Notably, the number of *ICS* in *G. hirsutum* did not double after allopolyploidization. Allopolyploidization (fusion of A and D genomes) is expected to double gene copies. However, over long-term evolution, some duplicates are lost due to functional redundancy to maintain dosage balance. Consistently, the numbers of *ADCS*, *ASA*, and *PAL* in tetraploid cotton are less than twice those in diploid cotton, indicating partial gene loss or selective elimination of redundant genes [39,40,41,42].

This structural conservation underscores the fundamental importance of these domains in maintaining the integrity of the SA biosynthesis pathway. In our motif analysis, we observed that some conserved motifs were partially lost in certain gene copies. We suggest that polyploidization and functional redundancy led to partial motif loss in some duplicated *PAL* and *ICS* genes, a common evolutionary process that helps balance genome dosage [43,44,45].

Promoter *cis*-element analysis revealed, as key genes for SA synthesis, that the stress response motifs showed significant enrichment, which aligns with the conserved core functions of SA in plant immunity (Figure 4 and Figure 5) [46]. Additionally, hormone response elements also exhibited significant enrichment, suggesting that plants require the synergistic action of phytohormones in complex environments, which serves as a crucial molecular basis for plants to balance growth and defense mechanisms [47].

Expression pattern analysis further showed that, overall, the genes in the ICS pathway had relatively stable expression levels across different tissues, stages of cotton fiber development, and stress treatments. This suggests that in cotton SA biosynthesis, the ICS pathway is mainly responsible for maintaining basal SA levels, consistent with the constitutive expression of *GhICS2*, while genes in the PAL pathway respond more strongly to various stresses, as multiple *GhPAL* genes are significantly upregulated under drought, salt, high temperature, low temperature, alkali stress, and Verticillium dahliae infection [48,49,50]. This indicates that the ICS pathway and the PAL pathway have distinct roles and functional differentiation in cotton SA synthesis and stress responses [17,51,52].

We identified distinct stage-specific expression patterns: *GhPAL1-2* exhibited peak expression at the fiber initiation stage, whereas *GhICS2-1* was most abundant during secondary wall deposition (Figure 6D,E). Both the fiber initiation stage and the secondary wall deposition stage are critical periods for cotton fiber development, which are associated with cotton fiber quality [26,53,54]. This significant expression difference highlights the nonredundant roles of the PAL pathway and the ICS pathway during cotton fiber development.

**Figure 7:**
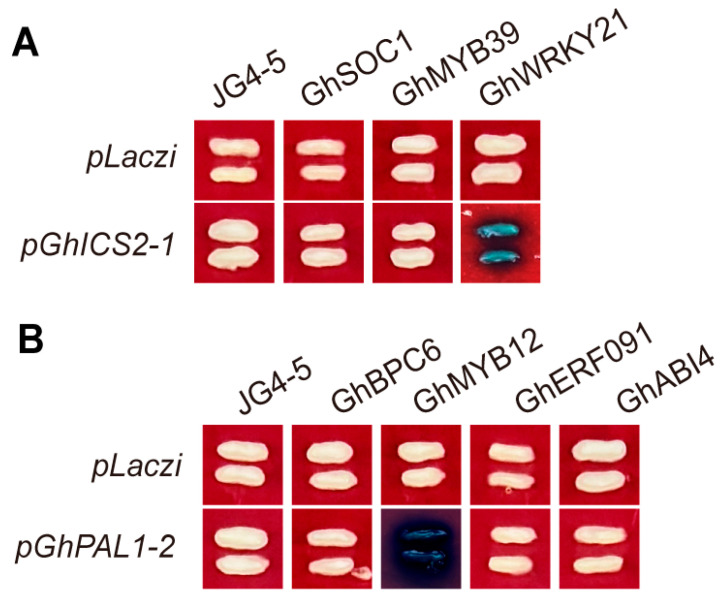
Analysis of Y1H results. (**A**) Y1H assays between the *GhICS2-1* promoter and three transcription factors. (**B**) Y1H assays between the *GhPAL1-2* promoter and four transcription factors. The empty vector pLaczi served as a negative control. Blue staining indicates positive protein–DNA interactions, confirming the binding of these TFs to the target promoters.

Y1H assays validated the direct binding of specific transcription factors to WRKY21 to the *GhICS2-1* and MYB12 to the *GhPAL1-2* promoters, providing a foundation for deciphering the upstream regulatory circuitry of SA biosynthesis in cotton (Figure 7). WRKY21 has been shown to be induced by SA, ABA, and JA, and functions as a negative regulator of ABA-mediated drought tolerance in cotton by facilitating the expression of GhHAB [55]. MYB12, an R2R3-MYB transcription factor belonging to subgroup 7, is a flavonol-specific activator of flavonoid biosynthesis that targets the promoters of key pathway genes, including PAL, CHS, and FLS [56,57]. Therefore, this result lays the foundation for subsequent studies on the upstream regulation of SA on cotton fiber development. Furthermore, we hypothesize that these transcription factors may regulate transcription by binding to these elements in the promoters, and whether the transcription factor plays a repressive or activating role needs to be further verified by dual-luciferase assays and EMSA experiments.

At the same time, we recognize the inherent limitations of this work; even so, they serve as a springboard for dissecting the remaining complexities of the SA pathway in cotton. Our analyses rely heavily on computational predictions and expression correlation analyses. While Y1H assays suggested potential interactions between transcription factors and promoters, functional validation is still required. We aim to validate these findings using reverse genetic methods, including gene knockout or overexpression, to pinpoint the exact functions of SA biosynthesis genes in cotton. Furthermore, profiling the dynamic expression patterns of SA-related genes across diverse growth conditions and stress treatments could offer novel insights into their functional regulation during plant immunity and abiotic stress responses. Building on this foundation, a comprehensive dissection of SA-mediated hormonal crosstalk and its biosynthetic transcriptional regulation is essential to define the specific roles of this phytohormone in major cropping systems.

## 4. Materials and Methods

### 4.1. Genome Database

The *A*. *thaliana* genome sequence and corresponding annotation files used in this study were derived from The *Arabidopsis* Information Resource (TAIR; https://www.arabidopsis.org, accessed on 20 January 2026) database. Genomic sequences, protein-coding sequences, and gene functional annotation files of four cotton cultivars, including *G*. *hirsutum* (NBI_v1.1), *G. arboreum* (A2_CRI), *G. barbadense* (Hai7124_ZJU) and *G*. *raimondii* (HAU_v1), were acquired from the Cotton Functional Genomics Database (CottonMD; https://yanglab.hzau.edu.cn/CottonMD, accessed on 20 January 2026) [58]. The genomic information of rice was derived from RAP (https://rapdb.dna.naro.go.jp/) [59]. All genome assemblies and annotation datasets were used for subsequent analyses, including gene identification, chromosomal localization, phylogenetic analysis, and collinearity analysis.

### 4.2. Identification and Characterization of SA Biosynthesis-Related Genes in Cotton

Sequences of SA biosynthesis-related genes collected from *A*. *thaliana* were from The *Arabidopsis* Information Resource (TAIR) database and used as reference queries to identify the key genes for SA biosynthesis in four cotton species. BLASTP (TBtools v2.210) searches (E-value: 1 × 10^−5^; % identity > 55) were performed against the protein databases of *G*. *hirsutum*, *G. arboreum*, *G. barbadense*, and *G*. *raimondii* using TBtools software (v2.210) [60]. Additionally, Hidden Markov Model (HMM) profiles of conserved SA-related domains (PF00221 for PAL and PF00425 for ICS) were obtained from the Pfam database and used for the identification of cotton proteomes using HMMER software (v3.3.2) [61]. Candidate genes identified by both approaches were integrated, redundant sequences were removed, and all retained candidates were verified for the presence of conserved domains to obtain the final set of the key genes for SA biosynthesis.

### 4.3. Phylogenetic Analysis of SA Biosynthesis-Related Genes

We performed phylogenetic analysis via MEGA X (v10.1.8) [62]. We aligned the SA protein sequences using the MUSCLE algorithm in MEGA X, applying default parameters. To select the best-fitting amino acid substitution model, we used the “Find Best DNA/Protein Models (ML)” module implemented in MEGA X. This function calculates the Bayesian Information Criterion (BIC) for each candidate model; the model with the lowest BIC score is considered the optimal substitution model for the given dataset [63]. Based on this analysis, the Jones–Taylor–Thornton (JTT) model with Gamma-distributed rate variation (+G) was identified as the best-fitting model. The phylogenetic tree construction was performed using the ML method, and the Jones–Taylor–Thornton (JTT) model with Gamma distribution (+G) was used for phylogenetic tree construction. We used 1000 bootstrap replicates to get branch support values. Tree visualization was achieved using the iTOL online tool (https://itol.embl.de, accessed on 20 January 2026).

### 4.4. Physicochemical Properties of SA Biosynthesis-Related Proteins

We predicted the physicochemical properties of PAL and ICS proteins, including MW, theoretical pI, and GRAVY, using TBtools (v2.210). Furthermore, the instability index and aliphatic index were computed to evaluate protein stability and hydrophobicity, respectively.

### 4.5. Chromosomal Localization of SA Biosynthesis-Related Genes

We mapped the chromosomal positions of *PAL* and *ICS* genes in four cotton species using available genomic annotation. Gene distribution on chromosomes was visualized using TBtools, and the results were used to analyze the potential association of *PAL* and *ICS* genes with chromosomal regions.

### 4.6. Sequence Analysis of SA Biosynthesis-Related Genes

Conserved motifs in the *PAL* and *ICS* genes were identified using the MEME Suite (v5.5.0) [64], with parameters set to a maximum of 10 motifs and an optimal motif width range of 6–100 amino acids. These motifs were annotated by comparing them with the InterPro (https://www.ebi.ac.uk/interpro, accessed on 20 January 2026) database to determine their functional significance. Domain architecture analysis was performed using TBtools software to identify characteristic functional domains within each subfamily. The coding sequences (CDS) of the *PAL* and *ICS* genes were analyzed to examine the gene structure and visualize the gene structures using TBtools (v2.210).

### 4.7. Analysis of Cis-Acting Elements of SA Biosynthesis-Related Genes in Cotton

We identified *cis*-elements in the promoter regions of key genes for SA biosynthesis, and 2000 bp promoter sequences of each gene were downloaded from the genome annotation files. *Cis*-acting elements were predicted using the PlantCARE database (http://bioinformatics.psb.ugent.be/webtools/plantcare/html/, accessed on 20 January 2026) [65]. The identified *cis*-elements were classified based on their functions, including stress response, hormone signaling, and developmental regulation. The distribution of *cis*-acting elements in the promoter regions was visualized using TBtools (v2.210) to analyze the patterns and potential functional relationships among the identified elements.

### 4.8. Collinearity Analysis of SA Biosynthesis-Related Genes in Cotton

Collinearity analysis of the key genes for SA biosynthesis in cotton was executed using TBtools software, with a particular emphasis on distinguishing between fragment, tandem, and genome-wide duplication signatures. Considering the polyploid nature of cultivated cotton, BLAST (TBtools v2.210) searches were systematically performed across the diploid A/D genome (*G. arboreum* and *G. raimondii*) and the corresponding A/D subgenomes of tetraploid species (*G. hirsutum* and *G. barbadense*). In an effort to capture both ancient and recent evolutionary events, duplicated gene pairs, including intraspecific pairs and interspecific combinations (*G. arboreum*-*G. barbadense*-*G. hirsutum-G. raimondii*), were utilized to construct collinearity diagrams. These syntenic maps, ultimately rendered by TBtools, served as the visual foundation for assessing the expansion mechanisms of these genes.

### 4.9. Expression Patterns Analysis of SA Biosynthesis-Related Genes in Cotton

Transcriptome datasets corresponding to the key genes for SA biosynthesis in *G*. *hirsutum* were obtained from the CottonMD database (https://yanglab.hzau.edu.cn/CottonMD, accessed on 20 January 2026) [58]. Publicly available RNA-seq data covering multiple tissues and developmental stages were downloaded and processed for subsequent expression profiling (NBI_v1.1, NCBI BioProject: PRJNA605345. SRA ID: SRX797886-SRX797920, SRX849508-SRX849561) [66]. The normalized expression values were used to evaluate tissue-specific transcriptional patterns and to assess differential expression among developmental stages.

### 4.10. Y1H Assay

Genomic sequences spanning approximately 2000 bp upstream of the translational start site of SA biosynthesis-related genes in *G*. *hirsutum* were amplified and subsequently cloned into the pLaczi reporter vector (Clontech, Mountain View, CA, USA), generating promoter–reporter constructs for use as bait in downstream assays. Candidate transcription factors predicted to regulate SA-related genes were selected, and their open reading frames were ligated into the JGY4-5 activation domain vector (Clontech) to generate effector constructs. Interaction screening was performed by introducing both reporter and effector plasmids into the yeast strain EGY48. Transformants were recovered on synthetic dropout medium lacking tryptophan and uracil to ensure successful plasmid incorporation. Surviving colonies were subsequently cultured on indicator plates containing X-gal for qualitative detection of reporter gene activation. Following incubation at 30 °C for 3–5 days, transcription factor–promoter associations were inferred from the appearance of blue staining, reflecting β-galactosidase enzymatic activity.

## Data Availability

All data generated or analyzed during this study are included in this article and its supplementary files. The datasets used in the present study are available from the corresponding author on reasonable request.

## Supplementary Materials

The following supporting information can be downloaded at: https://www.mdpi.com/article/10.3390/ijms27093936/s1.
