# Supplementary material for: Genome-Wide Identification and Characterization of the Key Genes for Salicylic Acid Biosynthesis in Four Cotton Species
Source: Int J Mol Sci. 2026 Apr 28;27(9):3936. doi: 10.3390/ijms27093936 (PMC13163499; doi:10.3390/ijms27093936)
Supplement: Supplementary file 1 [file ijms-27-03936-s001.zip › ijms-4222671-supplementary.pdf]

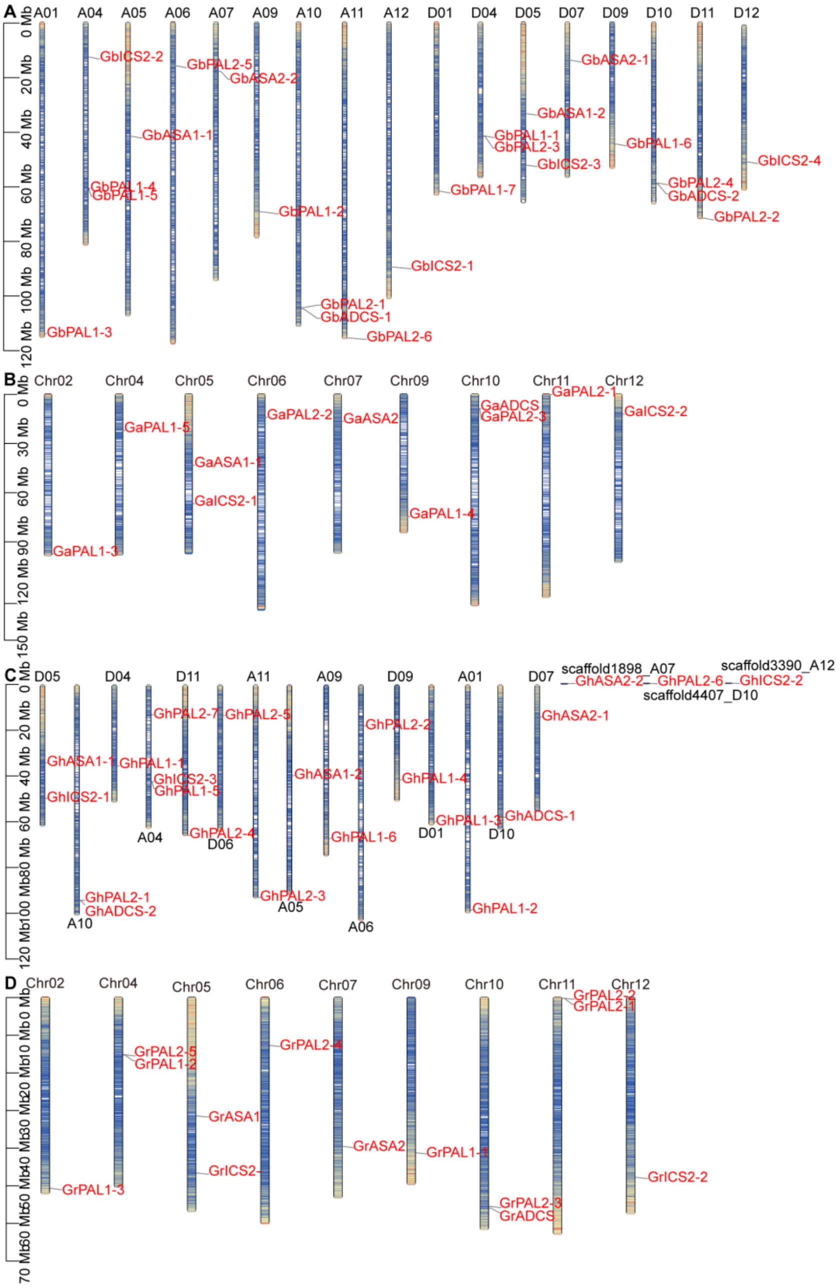

Supplementary Figure S1. Chromosomal distribution and location of SA biosynthesis-related genes: **(A)** Chromosomal localization of SA biosynthesis members from *G. barbadense* (Gb). **(B)** *G. arboreum* (Ga). **(C)** Chromosomal localization of SA biosynthesis members from *G. hirsutum* (Gh). **(D)** Chromosomal localization of SA biosynthesis members from *G. raimondii* (Gr). The vertical bar on the left represented the size of the chromosome, Mb, Mega base pair. The chromosomes number was located above or below each chromosome, only shows the chromosomes carrying the key gene for SA synthetase.

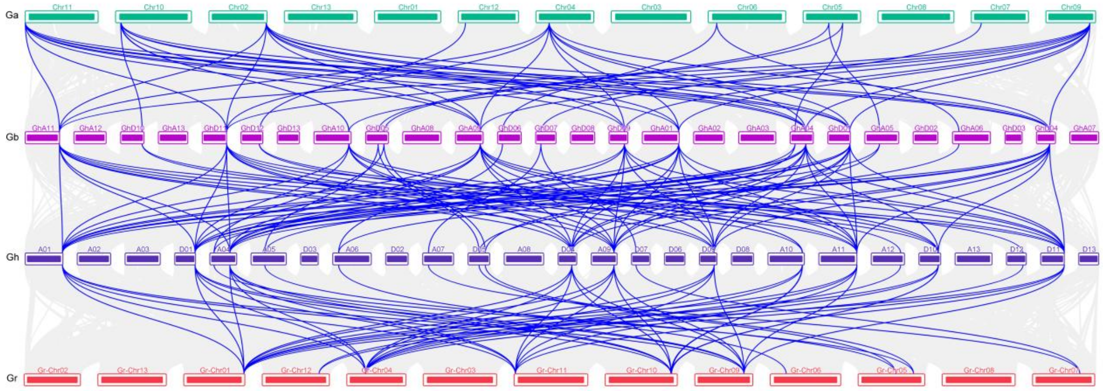

Supplementary Figure S2. The analysis of SA biosynthesis-related genes collinearity in *G. arboreum*, *G. barbadense*, *G. hirsutum* and *G. raimondii* , highlighted by blue lines.

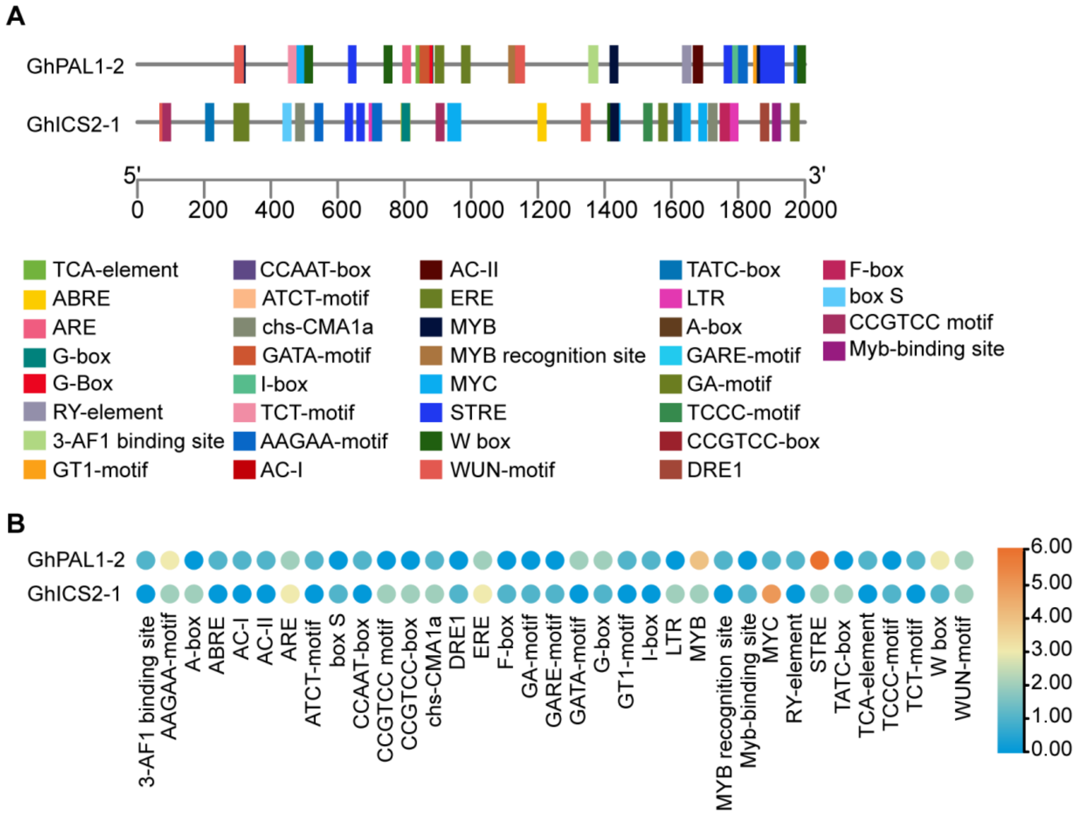

Supplementary Figure S3. *Cis*-element analysis of the *GhPAL1-2* and *GhICS2-1* promoters.: **(A)** *Cis*-acting regulatory elements analysis in the promoter regions of *GhPAL1-2* and *GhICS2-1*. The 2000 bp upstream sequences from the translation start codon (ATG) of GhPAL1-2 and GhICS2-1 were retrieved from the cotton genome database, and cis-acting elements were predicted using the PlantCARE database. Different colored boxes represent distinct types of cis-acting elements. The horizontal axis indicates the position relative to the translation start site (5' to 3'), and the length of the promoter region is 2000 bp. **(B)** Quantitative analysis of cis-acting regulatory elements in the promoter regions of *GhPAL1-2* and *GhICS2-1*. The heatmap illustrates the copy number of each type of cis-acting element identified in the 2000 bp promoter regions of GhPAL1-2 and GhICS2-1. The color gradient from blue to orange represents the increasing number of element copies (ranging from 0 to 6).

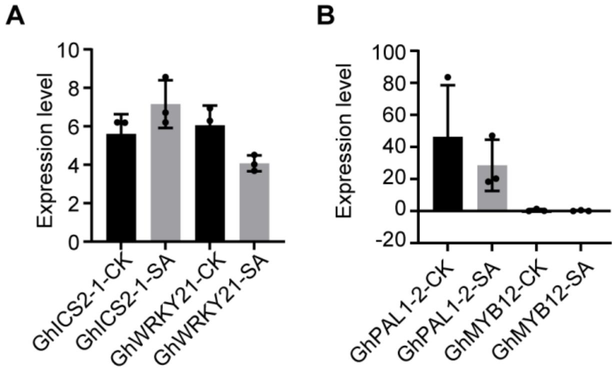

Supplementary Figure S4. Expression analysis of SA biosynthesis-related genes and transcription factors in cotton after exogenous SA treatment. **(A)** Relative expression levels of *GhICS2-1* and GhWRKY21 in cotton leaves under control (CK) and SA treatment conditions. **(B)** Relative expression levels of *GhPAL1-2* and GhMYB12 in cotton leaves under control and SA treatment conditions. The black bars represent the control group, and the gray bars represent the SA treatment group. Error bars indicate SD (n = 3)
